# Right-Sided Pyriform Sinus Fistula: A Case Report and Review of the Literature

**DOI:** 10.1155/2012/934968

**Published:** 2012-01-22

**Authors:** Rachel B. Cain, Peter Kasznica, William J. Brundage

**Affiliations:** Division of Otolaryngology, The University of Vermont, 111 Colchester Avenue, Burlington, VT 05401, USA

## Abstract

*Objectives*. Pyriform sinus fistulae arise from disturbances in the development of the fetal third and fourth branchial pouches and are predominantly found on the left side. We report the rare case of a right-sided pyriform sinus fistula presenting as a lateral neck abscess. *Study Design*. Case report. *Methods*. A 24-year-old woman presented with a two-week history of right-sided neck abscess. A fluoroscopic sinogram revealed a fistulous tract extending from the abscess to the apex of the right pyriform sinus. It was determined that the fistula was likely a third or fourth branchial remnant, a rare right-sided finding. Chemocauterization of the fistulous tract with 40% trichloroacetic acid was used to successfully treat the patient. *Results*. Approximately 93–97% of branchial pouch anomalies are left sided. Treatment options include surgical excision and cauterization. *Conclusions*. Branchial cleft cyst and pyriform sinus fistula must be considered in the diagnosis of cervical abscess in either side of the neck.

## 1. Introduction

Pyriform sinus fistulae are epithelialized tracts representing a malformation of the embryological third or fourth branchial pouch. While these fistulae present in many different ways, the vast majority are left sided. The theoretical and differing anatomical paths of third and fourth branchial fistulae have been widely described, but rarely seen clinically or captured radiographically. Treatment of these sinus tracts is also quite variable. We present a case of a rare right-sided pyriform sinus fistula demonstrated radiographically and treated successfully with chemocauterization.

## 2. Case Presentation

A previously healthy 24-year-old Caucasian woman presented to her primary care physician with right-sided odynophagia, sore throat, and subjective fevers following an upper respiratory infection. The patient was given an oral steroid course and azithromycin for presumed pharyngitis. Over the next four days she developed progressive neck swelling prompting her presentation to the emergency department. Due to worsening odynophagia and neck swelling, she was unable to tolerate solid foods.

On exam, the patient was noted to have tenderness, induration, and mild erythema along the right lateral neck. Flexible laryngoscopy revealed a normal larynx and upper aerodigestive tract, with no airway compromise or impingement. The patient had normal true vocal cord mobility bilaterally. Computed tomography with intravenous contrast was performed and showed a large cystic lesion with air and fluid extending from the right larynx laterally and inferiorly toward the thyroid. The patient underwent a needle decompression of the cystic mass, producing approximately 10 milliliters of purulent debris. She was admitted to the hospital for intravenous antibiotics and observation.

While in the hospital the patient improved clinically, but her cystic lesion reaccumulated fluid and ultimately necessitated decompression with a pigtail catheter drain placed by interventional radiology. The drain provided adequate decompression of the neck mass, allowing for improved swallowing ability.

**Figure 1:**
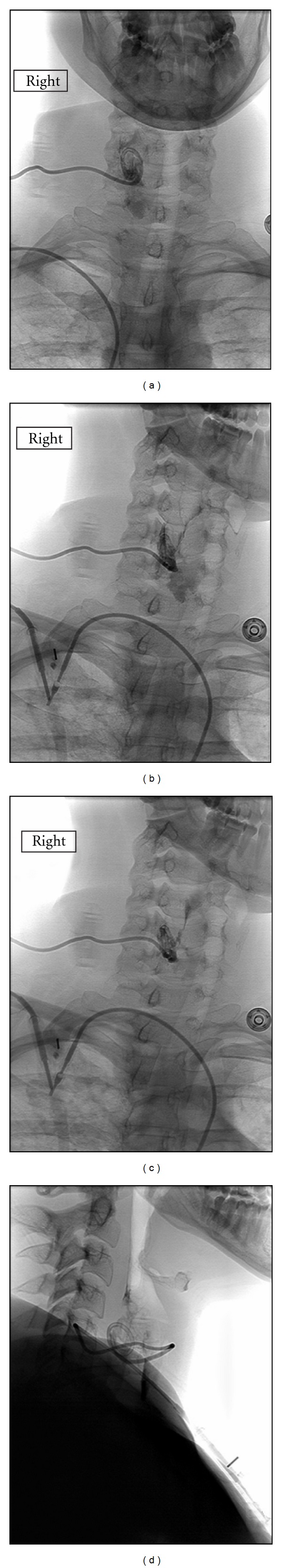
Fluoroscopic sinogram of pigtail catheter drain in the right lateral neck. (a) Anteroposterior view depicting pigtail catheter placed in the right cervical abscess. (b) Oblique view shows contrast exiting catheter and flowing into a fistulous tract leading to (c) the apex of the right pyriform sinus. (d) Lateral view.

During her hospital stay the patient underwent a fluoroscopic sinogram of the pigtail catheter drain and her cystic neck mass to further explore its origin (Figure 1). An aberrant connection of the cystic mass to the apex of the right pyriform sinus was discovered, suggesting the presence of a right third or fourth branchial cleft cyst and fistulous tract.

**Figure 2:**
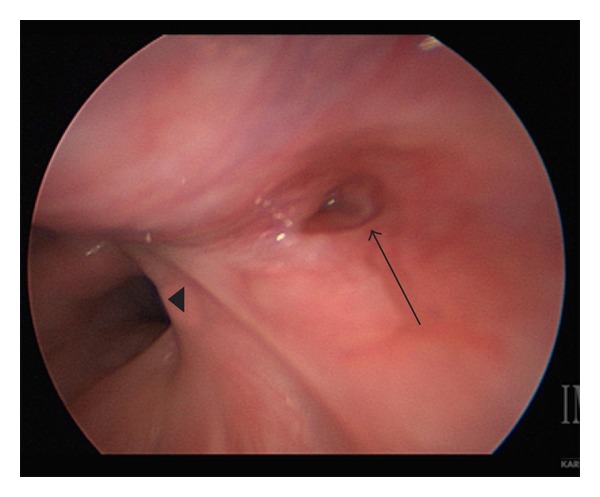
Fistulous tract (arrow) visualized in the right pyriform sinus adjacent to esophagus (arrowhead).

**Figure 3:**
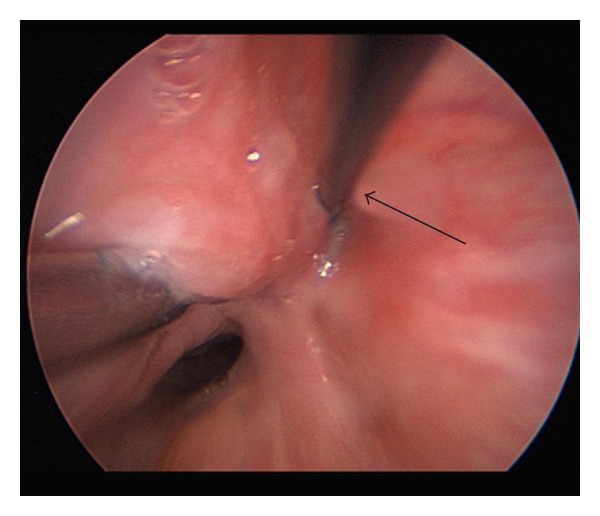
Right pyriform sinus fistulous tract cannulated with straight laryngeal probe (arrow).

The patient was discharged home with a prolonged course of oral clindamycin and a drain in place. She returned to clinic five days later with decreased drain output and significantly less turgor around the area of the neck mass. Two weeks later she was taken to the operating room for direct laryngoscopy, esophagoscopy (Figures 2 and 3), and chemocauterization of the fistulous tract with 40% trichloroacetic acid (TCA).

**Figure 4:**
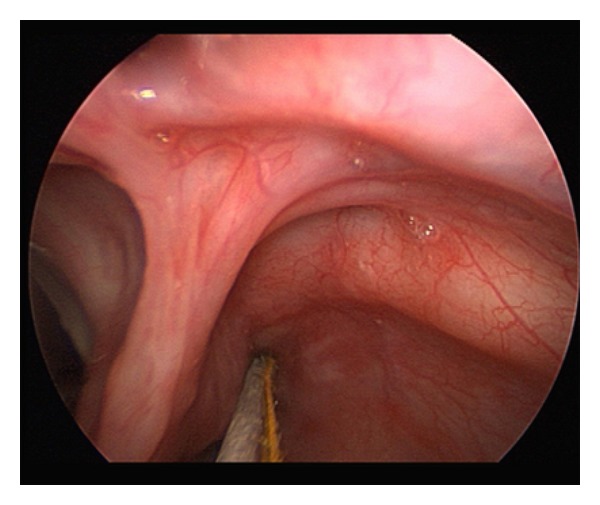
Laryngeal forceps used to insert TCA-soaked micropledget into fistulous tract.

**Figure 5:**
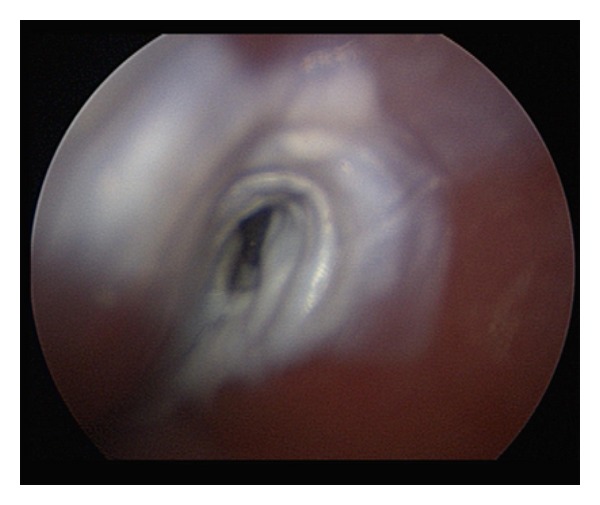
Ablated fistulous tract after cauterization with 40% TCA.

Micropledgets soaked in TCA were carefully placed into the fistulous tract (Figure 4). This was repeated for a total of three times, and the area was then rinsed with saline and inspected (Figure 5).

**Figure 6:**
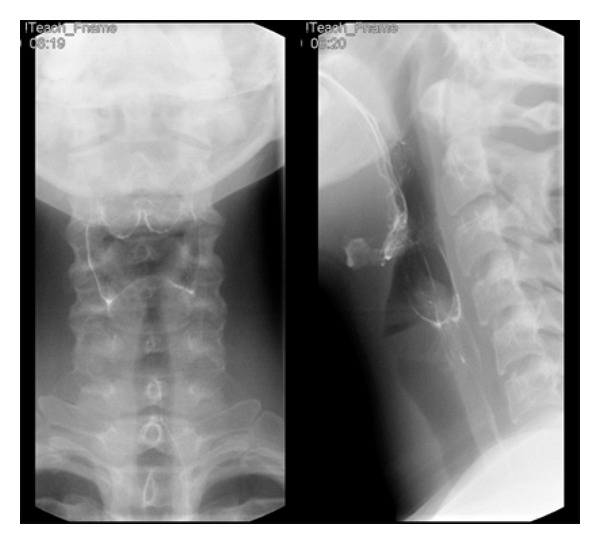
Esophagram (anteroposterior and lateral views) of cervical esophagus with barium swallow demonstrating a slight widemouthed outpouching of the right hypopharynx without contrast pooling. There is absence of a fistulous tract.

Postoperative examination ten days after laryngoscopy and cauterization revealed no evidence of fistula patency, and the patient's neck mass had largely resolved. Similar findings persisted in follow-up one month later. Repeat esophagram four months later confirmed fistula closure (Figure 6).

## 3. Discussion

Third and fourth branchial fistulae, also known as pyriform sinus fistulae, are epithelialized tracts connecting the skin of the neck to the foregut. These congenital anomalies arise from disturbances in the development of the fetal branchial apparatus. There are several hypotheses as to the specific origin of these fistulae. The classic description is based on an understanding of the segmental branchial anatomy and involves persistence of the pharyngobranchial duct, which connects the third and fourth pharyngeal pouches to the pharynx and normally degenerates during the seventh week of development. Persistence of this duct results in a sinus tract that communicates with the pyriform fossa, representing persistence of both branchial cleft and corresponding pouch [1, 2].

Third branchial fistulae are classically described as coursing from an anterior [3] and cephalad location in the pyriform fossa, piercing the thyrohyoid membrane, and tracking above the superior laryngeal nerve [1, 4]. These tracts then loop over the hypoglossal nerve, inferior to the glossopharyngeal nerve and posterior to the internal carotid artery [5].

Alternatively, classically described fourth pouch fistulae follow a more indirect route, coursing from the pyriform apex or proximal esophagus [1] below the superior laryngeal nerve, above the carotid bifurcation. They then descend in the tracheoesophageal groove parallel to the recurrent laryngeal nerve into the mediastinum, loop under the aortic arch on the left side and subclavian artery on the right side, and ascend posterior to the common carotid artery until exiting at the skin surface [1, 4, 5]. As with branchial anomalies of the second pharyngeal pouch, the external opening of both third and fourth pouch remnants arises at the same location in the skin overlying the anterior border of the sternomastoid muscle, which is the location of the embryologic cervical sinus.

While the proposed course of these fistulae is frequently quoted in the literature, differentiating third from fourth anomalies is often difficult, as there are some overlapping features, and precise identification of anatomic relationships at the time of diagnosis and treatment is not always possible [1]. These descriptions are infrequently seen in case reports, and no one has ever reported a lesion following the classical pathway of a fourth branchial fistula. This led James et al. [5]. to propose an alternative theory for the embryologic origin of third and fourth branchial fistulae, suggesting that they may actually be derivations of the embryologic thymopharyngeal duct, which forms as the thymus descends during fetal development.

Complete congenital third and fourth branchial fistulae are rare, with most presenting originally as sinus tracts before becoming secondary iatrogenic fistulae [5]. These lesions are most commonly observed in children and young adults. A recurrent lateral neck abscess (42%) and suppurative thyroiditis (45%) are the most common presentations [6], although upper respiratory infections, recurrent retropharyngeal abscess and cellulitis, odynophagia, stridor, and mediastinitis as presenting features have also been reported [1, 7–10]. Approximately 93–97% of fourth pouch anomalies are left sided [2, 3, 5, 6, 11] which may be related to the absence or involution of the ultimobranchial body on the right side, in addition to asymmetric vascular agenesis during fourth arch development [6, 9, 10]. Females are slightly more prone, and age at diagnosis ranges from birth to adulthood [1, 11].

Diagnosis of third and fourth branchial fistulae should be considered in any child or young adult with recurrent lateral cervical abscesses. In these instances, nasal fiberoptic endoscopy occasionally reveals a fistulous opening in the hypopharynx. Contrast-enhanced computed tomography may reveal abnormal soft tissue swelling with enhancement along the course of the tract, deformation of the involved pyriform fossa, cutaneous opening of the fistula, and/or gas along the course of the tract [9].

 Ultrasonography and CT imaging can be helpful in making a diagnosis or planning for treatment. Gas within the area of the left upper pole of the thyroid gland on ultrasound is considered pathognomonic of a pyriform fossa sinus [6].

Barium esophagography has also been used to demonstrate the hypopharyngeal fistulous opening with a sensitivity of up to 80% but is less useful in the acute inflammatory phase [1, 2]. In contrast, direct laryngoscopy often allows visualization of the fistulous opening in the pyriform fossa and can be performed during acute episodes [6]. When the sinus tract is visualized, the use of CT fistulogram can be used to delineate the lesion course, which can prevent the use of unnecessary surgical exploration [12].

Treatment should be preceded by the administration of appropriate antibiotics in order to allow regression of associated inflammation [7]. Surgical excision is considered definitive therapy and can be aided by internal or external cannulation of the fistula with a Fogarty catheter. If the fistula or sinus courses through the thyroid gland, lobectomy should be performed [4, 10]. However, surgical treatment poses significant risk to the recurrent laryngeal nerve, especially in the setting of scarring and fibrosis from recurrent abscesses.

Chemocauterization with 40% trichloroacetic acid (TCA) with or without primary mucosal coverage is a less invasive treatment modality, though it may be prone to recurrence [12, 13]. Electrocauterization of the sinus tract has also been used. The advantage of TCA over electrocautery is that a longer segment of the fistulous tract can be obliterated depending on how deeply the acid penetrates into the sinus [14] and there is less risk to adjacent structures from heat and electrical spread. When compared to surgical management, this minimally invasive technique avoids the risk of an open surgical procedure allowing for less morbidity and earlier hospital discharge [14].

 Chemocauterization can even be repeated several times without making later surgery more difficult if required. However, in cases of recurrence after conservative treatment, surgery is recommended [6].

Branchial cleft cyst and pyriform sinus fistula must be considered in the diagnosis of cervical abscess in either side of the neck, despite the rarity of right-sided lesions. Chemocauterization is a safe and effective first-line treatment.
